# MAKING SENSE OF A DISEASE THAT MAKES NO SENSE: CULTURAL UNDERSTANDINGS OF DEMENTIA AMONG ALASKA NATIVE CAREGIVERS

**DOI:** 10.1093/geroni/igae098.0893

**Published:** 2024-12-31

**Authors:** Jordan Lewis

**Affiliations:** University of Alaska Fairbanks, College of Indigenous Studies, Fairbanks, Alaska, United States

## Abstract

With the rate of Alzheimer’s disease and related dementias (ADRD) increasing among Alaska Indian/Alaska Native (AI/AN) people, the Alaska Native Health system is ill-prepared to meet the challenges associated with the growing population at risk of ADRD. The high cost of care, inadequate training of health care providers, and lack of supportive services for caregivers are especially concerning. Interviews were conducted with 22 AN caregivers for ANs with ADRD in communities across Alaska. Interviews lasted approximately 60 min and were transcribed verbatim. We employed directed content analysis to examine the major agreements and disagreements between the participants’ understandings of ADRD in each of the domains of Kleinman’s Explanatory Model of Illness. Caregivers and health care providers expressed concerns about the lack of understanding, resources, and awareness of ADRD among ANs in rural and urban communities. Both caregivers and providers recognized the need to obtain an early diagnosis, blend Western and traditional medicines, promote lifestyle and dietary changes, and foster training for caregivers. Health care providers acknowledged their limited exposure to AN understanding of ADRD and wish to receive culturally relevant training to better serve AN. As the older AN adult population grows, the need for culturally responsive training and support services will continue to increase. We recommend establishing rural outreach and support groups for caregivers, developing an understanding of how ANs view ADRD to train and educate health care providers, and implement screening early for memory loss during routine medical examinations

